# Supplementary material for: Vibrational control of selective bond cleavage in dissociative chemisorption of methanol on Cu(111)
Source: Nat Commun. 2018 Oct 2;9:4039. doi: 10.1038/s41467-018-06478-6 (PMC6168487; doi:10.1038/s41467-018-06478-6)
Supplement: Supplementary file 1 — Supplementary Information [file 41467_2018_6478_MOESM1_ESM.pdf]

Supplementary Information for

Vibrational control of selective bond cleavage in dissociative chemisorption of methanol on Cu(111)

Chen *et al.*

Supplementary Methods

Density functional theory (DFT) calculations. The total energy calculations and geometry optimizations were performed by means of using the Vienna Ab initio Simulation Package (VASP).[1,2] We modeled the Cu(111) surface with a 3×3 unit cell and four Cu layers, with a vaccum space of 20 Å between periodic slabs and a dipole correction in the *Z* direction. The generalized gradient approximation (GGA) was used to treat the electron exchange-correlation effects,[3] with the optPBE-vdw functional[4] to correct the van der Waals effect. The interaction between the ionic cores and electrons was represented by the projector-augmented wave (PAW) method.[5] The kinetic energy cutoff of plane waves was 400 eV. The first Brillouin zone was sampled by using Monkhorst-Pack scheme with a 5×5×1 *k*-points grid mesh.[6] The calculated lattice parameter for bulk Cu was 3.635 Å in good agreement with the experimental value of 3.615Å.[7] We chose final parameters with a balance of computational costs and accuracy. Stationary points were optimized by the conjugate gradient algorithm and the transition states were searched by means of the dimer method.[8] Transition states were verified by the presence of a single imaginary frequency.

Potential energy surface. Following our earlier work,[9-11] the permutation invariant polynomial-neural network (PIP-NN) approach was used to fit the potential energy surface (PES). This method has been successfully applied to both gaseous and gas-surface systems.[12,13] Utilizing symmetry functions as the input layer of the NN,[14,15]

which are constructed by permutation invariant polynomials (PIPs)[16] of primitive symmetry functions, PIP-NN function guarantees both intrinsic surface and permutation symmetry in the system.[9]

The primitive symmetry functions are expressed in terms of the Cartesian coordinates according to the periodicity of Cu(111) and the internal coordinates of the molecule,

$$G_{1\text{-}15} = \exp(-\lambda \mathrm{r}_{ij}), \tag{1}$$

$$G_{13+3i} = \left[\cos(\frac{4\pi Y_i}{a\sqrt{3}}) + 2\cos(\frac{2\pi X_i}{a})\cos(\frac{2\pi Y_i}{a\sqrt{3}}) \right] \times G_{15+3i}, \tag{2}$$

$$G_{14+3i} = \left[\sin(\frac{4\pi Y_i}{a\sqrt{3}}) - 2\cos(\frac{2\pi X_i}{a})\sin(\frac{2\pi Y_i}{a\sqrt{3}}) \right] \times G_{15+3i}, \tag{3}$$

$$G_{15+3i} = \exp(-\lambda z_i), \tag{4}$$

$$G_{34} = \left[\cos(\frac{4\pi Y_{CM}}{a\sqrt{3}}) + 2\cos(\frac{2\pi X_{CM}}{a})\cos(\frac{2\pi Y_{CM}}{a\sqrt{3}}) \right] \times \exp(-\lambda Z_{CM}), \tag{5}$$

$$G_{35} = \left[\sin(\frac{4\pi Y_{CM}}{a\sqrt{3}}) - 2\cos(\frac{2\pi X_{CM}}{a})\sin(\frac{2\pi Y_{CM}}{a\sqrt{3}}) \right] \times \exp(-\lambda Z_{CM}), \tag{6}$$

$$G_{36} = \exp(-\lambda Z_{CM}), \tag{7}$$

where a is the lattice constant and λ is usually taken the value of 1.0 Å^{-1} and X_{CM}, Y_{CM}, Z_{CM} denote the coordinate for center of mass of CH_3OH at x, y, z direction, respectively. G_1 to G_{15} are exponential functions of 15 internuclear distances in methanol. G_{16} to G_{33}

are Fourier expansions of atomic lateral coordinates (x_i,y_i) reflecting the Cu(111) periodicity and exponential functions of z_i ensuring the flatness of the asymptotic potential when the molecule is far away from the surface,[10] where i=1,2,3,4,5,6 denote H, H, H, H, O and C atoms, respectively. The permutation symmetry owing to the four identical H atoms is then adapted by constructing PIPs from G_1-G_{33} (G_{34}-G_{36} themselves are permutation invariant already). Using the computational algebra software SINGULAR,[17] 546 PIPs up to the sixth degree were found as the minimum generating set of PIPs for this system. These terms plus G_{34}-G_{36}, were used in the input layer of PIP-NN.

To sample the configuration space, we first run hundreds of ab initio molecular dynamics (AIMD) trajectories which start at the positions close to transition states. A primitive PES was fitted with the geometries and energies extracted from the AIMD trajectories. In order to reduce the oversampling in the entrance channels, the geometric criteria based on the root mean square error (RMSE) of the Euclidean distance between two points was used to avoid the inclusion of points that were too close to each other. Additional points were obtained via running classical trajectories with a variety of initial conditions on the primitive PES focusing on the interaction and product regions. Based on our earlier method,[17] we added a new point to the data set only if it satisfies both the distance and energy criteria. The process was carried out iteratively and the selection criterion was adapted with results of QCT calculations. For example, the criterion of Euclidian distance was varied from 0.5 to 0.2 Å, and of energy from 0.2 and

0.05 eV, with increasingly more data points. Eventually, the PES was considered to be converged when the reaction probability curve at a given nozzle temperature remained unchanged with the increasing number of points. More detailed descriptions of the PIP-NN fitting and sampling strategy are available in our earlier works.[9-11]

In total, 204538 DFT points were collected for CH_3OH/Cu(111) system. Extra 6000 free CH_3OH structures were computed whose energy plus the energy of the bare surface were taken as correct description of the asymptotic region. The whole data set is divided randomly into two subsets, i.e. training (90%) and testing (10%) sets, with the former being used to optimize the parameters and the later to validate the fit. The NN structure consists of 18 and 100 neurons in the two hidden layers, respectively, denoted as 549-18-100 with total 11901 parameters. A weighting function given by $w_i = \left(\bar{E} / \left(E_i + \bar{E}\right)\right)^3$ with $\bar{E}$ =2.0 eV for the *i*th point whose energy exceeds $\bar{E}$, was employed to ensure that higher energy points have lower weights. The NN weights and biases were iteratively optimized using our recently developed hybrid extremely learning machine and Levenberg-Marquart algorithm.[18] The ultimately converged PES is an average over the three best fits, leading to the overall RMSE of 37.2 meV.

Quasi-classical trajectory method. Quasi-classical trajectory (QCT) calculations were performed with a heavily modified VENUS version.[19] To that end, the CH_3OH molecule was initiated at 10.0 Å above the metal surface, with its lateral coordinates randomly sampled in the unit cell. The initial incident angle (θ_i) with respect to surface

normal was fixed to 0°. To check the convergence with respect to the number of DFT points, the ro-vibrational momenta were sampled on the basis of Boltzmann distribution at a corresponding nozzle temperature T_N via a Monte-Carlo scheme. In the state-specific calculations, rotational energy was fixed at zero and the displacement coordinates and vibrational momenta were sampled by normal modes. The integration time step was set as 0.10 fs and almost all trajectories were conserved within 0.05 meV. A trajectory was considered as reactive if the O-H/C-H/C-O bond distance exceeds 2.2/2.2/2.7Å and as scattered if the center of mass of CH_3OH molecule reached 10.1 Å above the surface with the velocity pointing away from the surface. These parameters were tested to converge the reaction probability.

Sudden Vector Projection Model. The Sudden Vector Projection (SVP) model[20] has proven to reasonably predict the mode specificity and bond selectivity in gas phase and gas-surface reactions.[17,21] It is based on the hypothesis that the timescale of the collision is too short for any internal energy redistribution within the reactant. An initial state having a large coupling with the reaction coordinate at the transition state is thus expected to give rise to a large vibrational efficacy. This coupling, can be quantified by the overlap, or equivalently, the alignment between the corresponding normal mode vectors. In practice, the molecular structure is optimized in the asymptote followed by a normal mode analysis to obtain the reactant vectors. The same procedure is done at the transition state whose structure is reoriented to have a maximal overlap with the reactant. The SVP values are then computed by projecting a reactant normal mode (or

translational) vector $\mathbf{Q}_i$ onto the reaction coordinate $\mathbf{Q}_{RC}$ of the transition state corresponding to the imaginary frquency, namely $p_i = \mathbf{Q}_i \cdot \mathbf{Q}_{RC}$. SVP model is essentially an extension of Polanyi's rules[22] to a multidimensional PES that is applicable to polyatomic reactions. Recent AIMD studies have demonstrated that sudden approximation is valid in a direct reaction with a remakable barrier, such as methane dissociation on metal surfaces.[23] As a result, SVP model is expected to work well in the present system.

Supplementary Discussion

Convergence tests of the molecular adsorption and activation energy for the CH_3OH/Cu(111) system are listed in Supplementary Table 1. Note that in these calculations the top two metal layers were relaxed. It is clearly shown that the 5×5×1 *k*-points and 400 eV kinetic energy cutoff of plane wave basis converge the energetics within ~0.05 eV, except for the co-adsorbed products. It should be noted that the energies of the co-adsorbed products are not important for our purpose as our PES would not cover such a large separation between products.

In Supplementary Table 2 and 3, we compare the optimized geometries and the frequencies of the stationary and saddle points along the minimum energy paths for multiple CH_3OH dissociation channels obtained from DFT and PES. It is clear that the PES well reproduces these features including the bond lengths and angles, energies, as well as harmonic frequencies.

Supplementary Fig. 1 compares the dissociation probabilities for O-H, C-H, and C-O bonds computed with various fits with different number of neurons and points at the same nozzle temperature (T_N=2000K) as a function of incidence energy. To check the convergence of the PES with respect to the number of points, we randomly removed up to 30000 points. There are no dramatic differences between dissociation probabilities with the removal of 10000 points and original points. However, a relatively larger change of reaction probability is observed if 30000 points were deleted. On the other hand, decreasing the number of neurons in the first hidden layer has little effect on the PES. Except some inevitably small fluctuations owing to statistical errors, these results indicate that our final fit, labelled as 549-18-100-204538, was well converged. Supplementary Fig. 2 shows the distributions of data points as a function of their energies and the fitting errors. It is clear that our selection of points covers a wide energy range of interest and most points have the fitting errors below 0.05 eV (the uncertainty of DFT calculations based on our convergence tests)

Supplementary Table 4 lists vibrational efficacies for all stretching modes of the CH_3OH molecule. Exciting the stretching mode of each type of bond, would almost exclusively prompt the cleavage of the corresponding bond while have a limited impact on others. These results are in qualitative agreement with the SVP values listed in Supplementary Table 5.

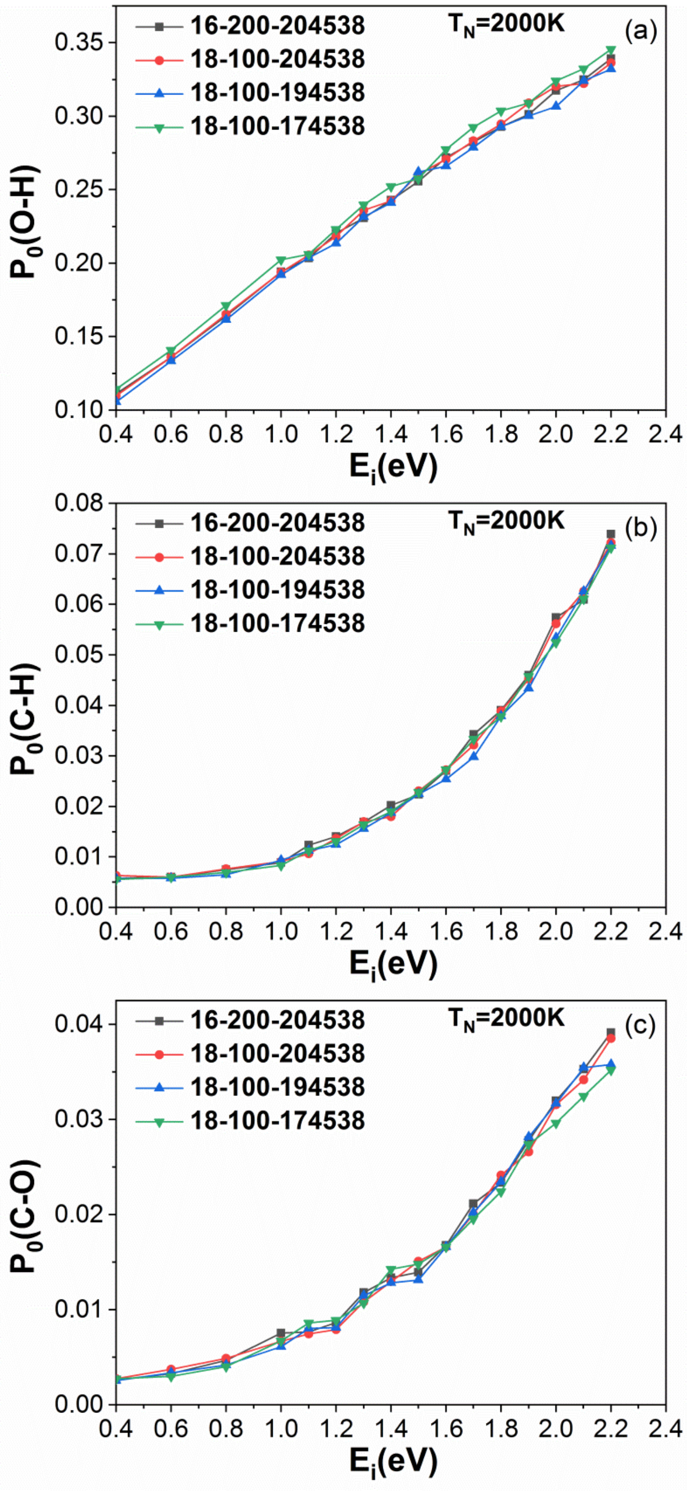

Supplementary Fig. 1 Convergence of dissociation probabilities. Comparison of O-H **(a)**, C-H **(b)**, and C-O **(c)** dissociation probabilities at nozzle temperature (T_N) of 2000 K obtained from various NN fits.

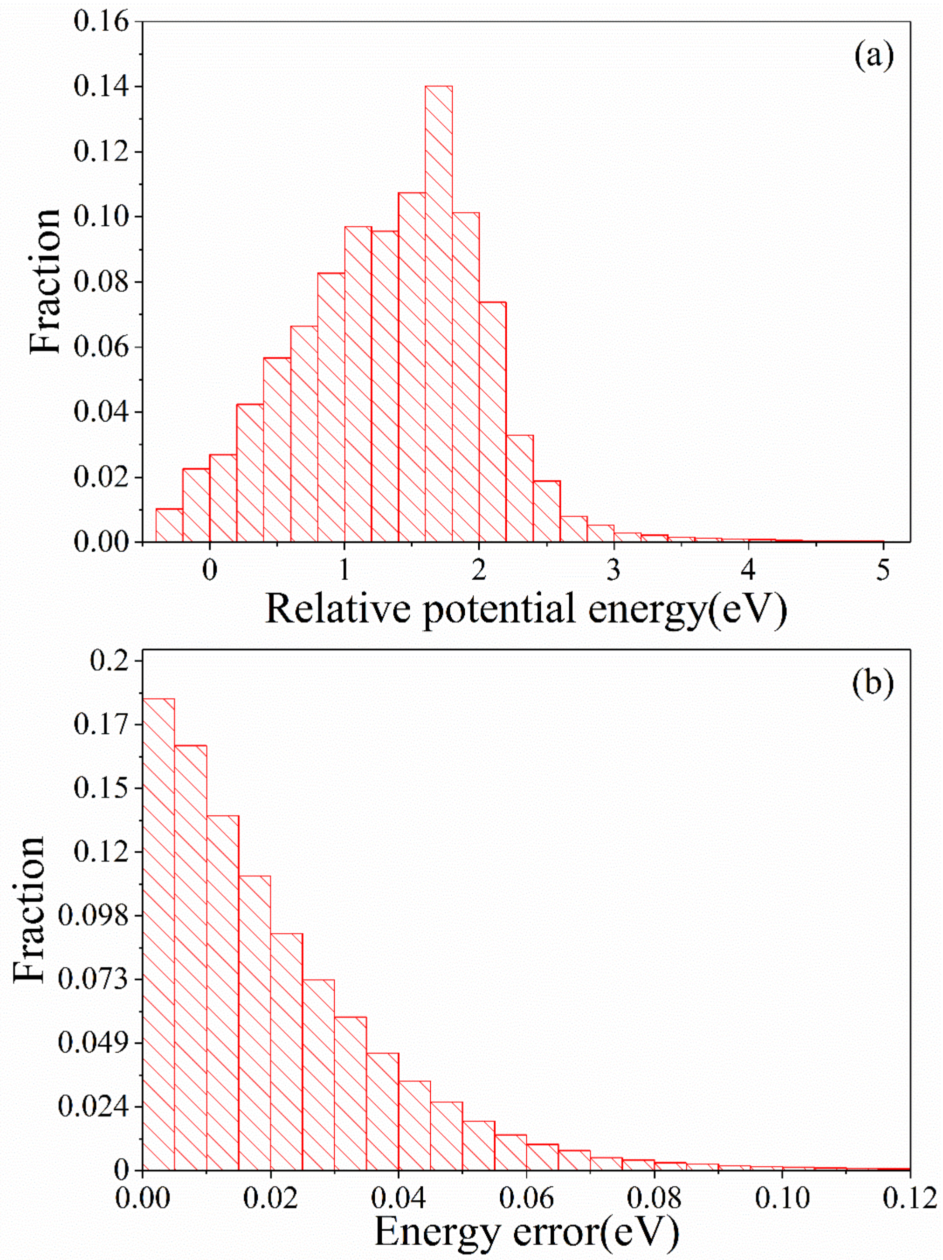

Supplementary Fig. 2 Analysis of data distributions. Distributions of **(a)** potential energies and **(b)** energy errors of DFT points in the data set.

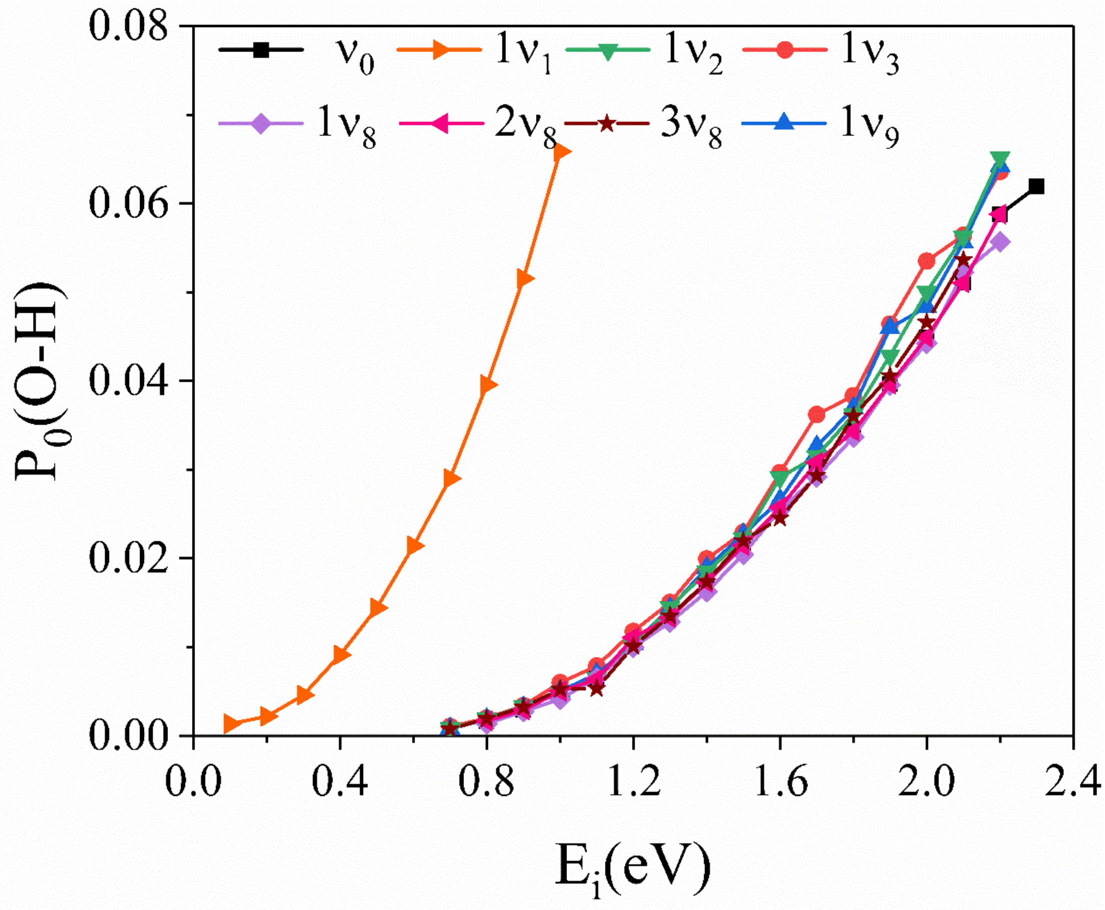

Supplementary Fig. 3 O-H dissociation. Dissociation probabilities as a function of incidence energy for several initial vibrational states.

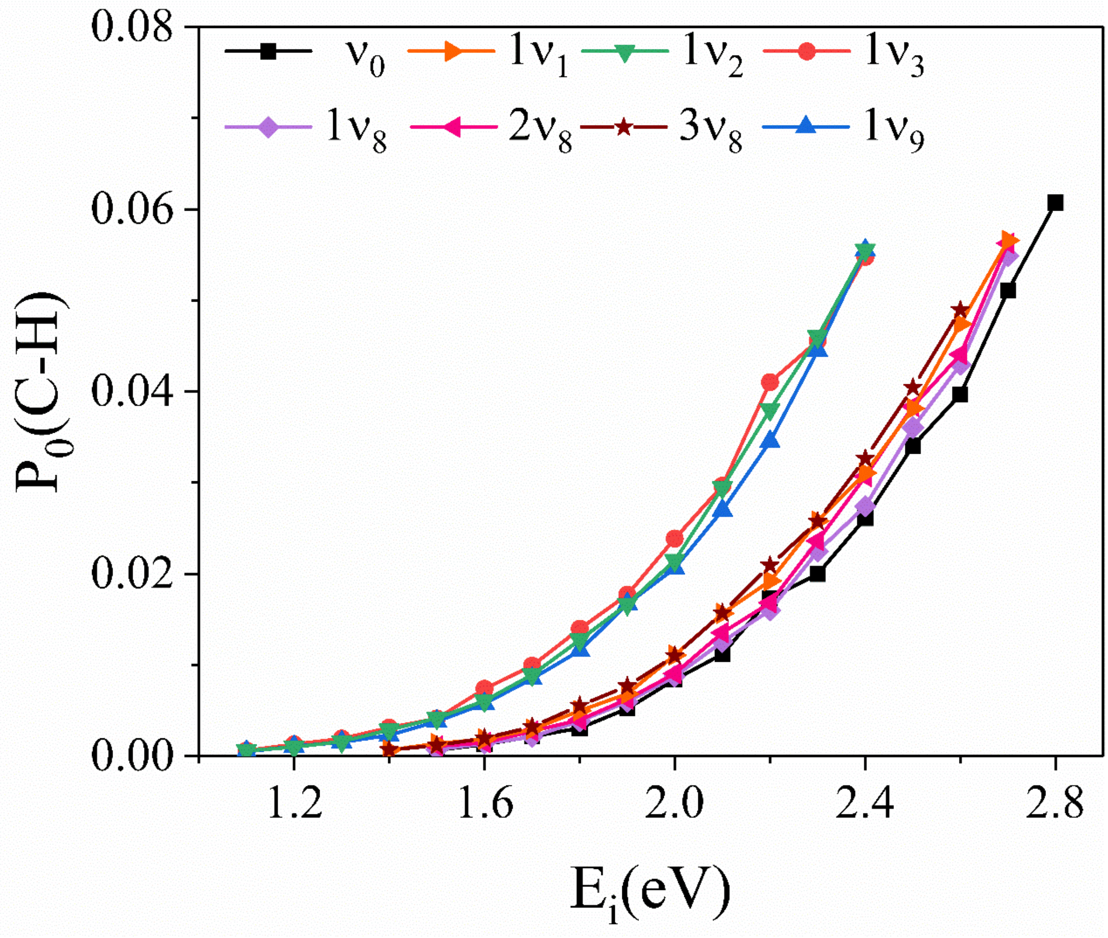

Supplementary Fig.4 C-H dissociation. Same as Supplementary Fig. 3 except for C-H dissociation probabilities.

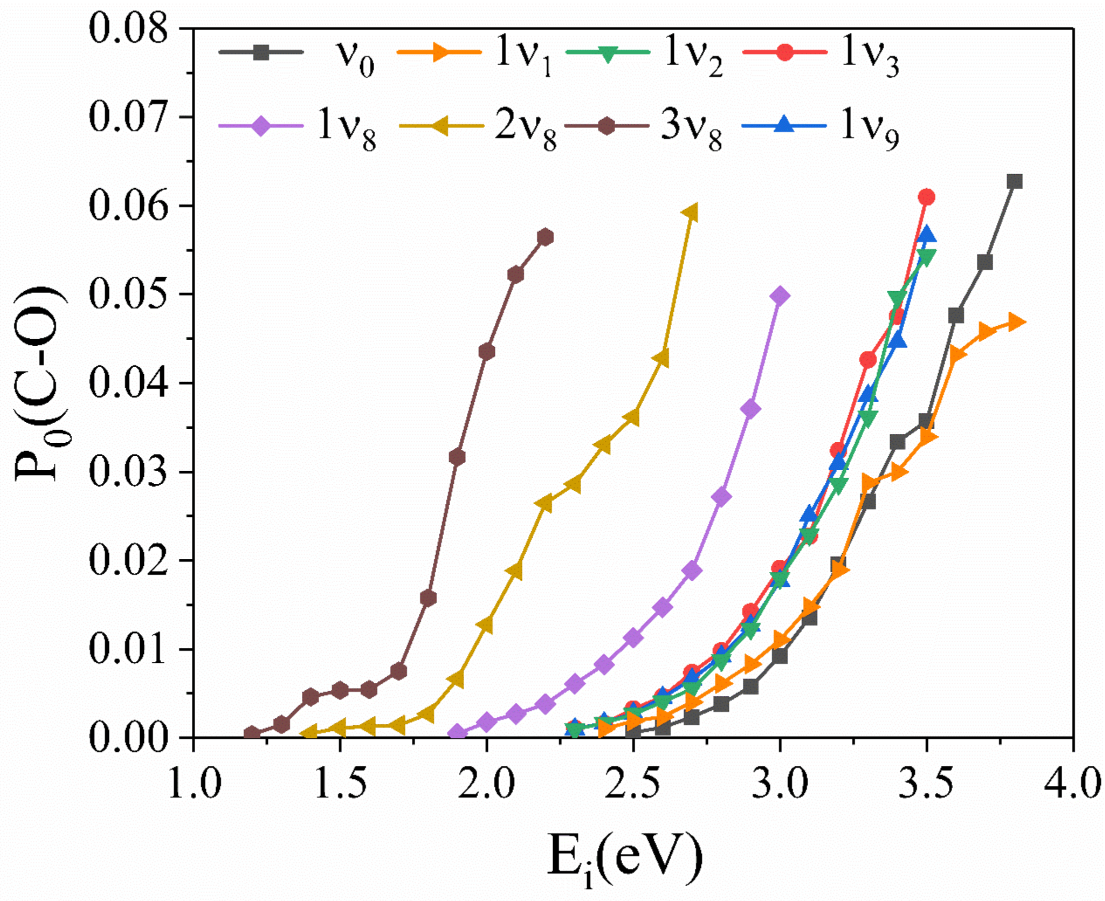

Supplementary Fig. 5 C-O dissociation. Same as Supplementary Fig. 3 except for C-O dissociation probabilities.

Supplementary Table 1. Convergence tests of energetics (in eV) with respect to the setup of the slab model. The adsorption energy is defined as $E_a = E_{mole+slab} - E_{mole} - E_{slab}$, where $E_{mole+slab}$, E_{mole}, and E_{slab} are energies of the binding structures, gaseous molecule, and bare surface, respectively. The activation energy is defined as $E_b = E_{TS} - E_{mole} - E_{slab}$. The reaction energy ΔE is defined as product energy minus E_{mole} and E_{slab}. Note that in these calculations the top two metal layers were relaxed.

Setups	E_a	E_b			ΔE		
		TS1[a]	TS2[a]	TS3[a]	PR1[a]	PR2[a]	PR3[a]
Energy cut:350eV k points: 3×3×1	-0.416	1.014	1.383	1.695	-0.253	0.862	-0.007
Energy cut:350eV k points: 5×5×1	-0.430	0.689	1.049	1.375	-0.495	0.651	-0.322
Energy cut:400eV k points: 3×3×1	-0.419	0.812	1.181	1.491	-0.246	0.860	-0.023
Energy cut:400eV k points: 5×5×1	-0.445	0.689	1.042	1.345	-0.496	0.646	-0.344
Energy cut:400eV k points: 7×7×1	-0.423	0.733	1.099	1.394	-0.415	0.715	-0.264
Energy cut:500eV k points: 5×5×1	-0.440	0.692	1.041	1.340	-0.498	0.648	-0.347

[a]The three dissociation channels correspond to $CH_3OH \xrightarrow{TS1} CH_3O+H$ (PR1), $CH_3OH \xrightarrow{TS2} CH_2OH+H$ (PR2), and $CH_3OH \xrightarrow{TS3} CH_3+OH$ (PR3), respectively.

Supplementary Table 2. Comparison of the structures parameters for species obtained on the PIP-NN PES and from DFT calculations. The distances are in Å, angles in degree, and energies in eV. Reference zero point energy is defined as CH_3OH + Cu(111) asymptote.

Structure	Geometry /Energy	Method	
		DFT	PES
Gaseous CH_3OH	r_{CH1}	1.103	1.102
	r_{CH2}	1.097	1.096
	r_{CH3}	1.103	1.102
	r_{OH4}	0.973	0.974
	r_{CO}	1.440	1.437
	θ_{COH4}	108.37	108.34
	Energy	0.000	-0.003
Physisorbed CH_3OH	r_{CH1}	1.111	1.103
	r_{CH2}	1.097	1.096
	r_{CH3}	1.100	1.101
	r_{OH4}	0.979	0.980
	r_{CO}	1.453	1.451
	Z_O	2.386	2.420
	Z_C	3.240	3.237
	θ_{COH4}	109.18	109.31
	Energy	-0.422	-0.419
TS1	r_{CH1}	1.102	1.100
	r_{CH2}	1.103	1.102
	r_{CH3}	1.104	1.102
	r_{OH4}	1.509	1.475
	r_{CO}	1.439	1.445
	Z_O	1.798	1.799
	Z_C	3.115	3.093
	θ_{COH4}	115.81	119.29
	Energy	0.791	0.794
TS2	r_{CH1}	1.090	1.089
	r_{CH2}	1.973	1.965
	r_{CH3}	1.094	1.094
	r_{OH4}	0.986	0.985
	r_{CO}	1.370	1.365

	Z_O	2.823	2.881
	Z_C	2.413	2.435
	θ_{OCH2}	108.17	105.76
	Energy	1.116	1.125
TS3	r_{CH1}	1.086	1.085
	r_{CH2}	1.087	1.088
	r_{CH3}	1.086	1.085
	r_{OH4}	0.987	0.983
	r_{CO}	2.320	2.366
	Z_O	1.610	1.613
	Z_C	2.913	2.929
	θ_{COH4}	104.32	106.99
	Energy	1.322	1.343

Supplementary Table 3. Same as Supplementary Table 2 except for normal mode frequencies in cm^{-1}.

Configuration	Note	Frequency
Gaseous CH_3OH	DFT	3704,3041,2964,2919,1473,1461, 1440,1335,1140,1051,993,286
	PES	3670,2975,2939,2887,1467,1463, 1432,1337,1111,1062,990,264
Physisorbed CH_3OH	DFT	3609,3041,2999,2936,1461,1453,1425,1312,1131, 1041,953,390.166,122,99,66,52,34
	PES	3596,3031,2993,2924,1472,1464,1427,1287,1113 1048,956,367,152,113,89,62,47,15
TS1	DFT	2982,2966,2909,1447,1443,1408,1121,1112,973, 947,500,249,162,152,87,61,3,-1096
	PES	3024,3017,2947,1470,1463,1426,1115,1101,979, 934,404,245,169,98,80,56,44,-1250
TS2	DFT	3461,3126,3004,1444,1334,1217,1134,1068,921, 685,649,503,256,189,124,79,48,-511
	PES	3565,3203,3073,1466,1319,1156,1153,1006,884, 641,627,476,263,174,129,60,36,-580
TS3	DFT	3498,3212,3195,3036,1385,1371,814,621,534, 464,431,278,189,139,83,70,49,-372
	PES	3620,3256,3226,3109,1419,1360,829,605,480, 445,398,286,159,133,84,67,59,-351

Supplementary Table 4. Calculated vibrational efficacies as a function of the probability of various vibrational modes in the O-H/C-H/C-O dissociation.

Dissociation Probabilities of O-H	0.001	0.005	0.01	0.02	0.03	0.04	0.05
ν_1	1.62	1.64	1.72	1.97	2.22	2.45	2.66
ν_3	0.22	0.21	0.19	0.22	0.26	0.31	0.35
ν_9	0.21	0.14	0.10	0.11	0.15	0.21	0.26
ν_2	0.29	0.17	0.11	0.11	0.15	0.21	0.26
ν_8	0.38	0.22	0.03	-0.07	-0.06	0.00	0.06
$2\nu_8$	0.20	0.19	0.10	0.04	0.05	0.07	0.10
$3\nu_8$	0.20	0.11	0.03	0.01	0.04	0.09	0.14
Dissociation Probabilities of C-H	0.001	0.005	0.01	0.02	0.03	0.04	0.05
ν_1	0.10	0.18	0.17	0.17	0.16	0.16	0.16
ν_3	1.10	1.00	1.01	1.01	1.01	1.01	1.01
ν_9	1.00	0.80	0.84	0.87	0.90	0.92	0.94
ν_2	0.95	0.86	0.89	0.92	0.94	0.95	0.97
ν_8	0.28	0.07	0.11	0.18	0.23	0.28	0.33
$2\nu_8$	0.33	0.14	0.15	0.18	0.20	0.23	0.25
$3\nu_8$	0.21	0.27	0.25	0.25	0.24	0.24	0.24
Dissociation Probabilities of C-O	0.001	0.005	0.01	0.02	0.03	0.04	0.05
ν_1	0.38	0.18	0.15	0.06	-0.04	-0.18	-0.37
ν_3	0.79	0.67	0.57	0.53	0.55	0.61	0.68
ν_9	0.75	0.60	0.53	0.49	0.50	0.53	0.57
ν_2	0.71	0.53	0.46	0.44	0.46	0.50	0.56
ν_8	5.13	5.00	4.21	4.15	4.44	4.82	5.25
$2\nu_8$	4.45	4.33	4.31	4.26	4.21	4.16	4.12
$3\nu_8$	3.57	3.37	3.45	3.76	4.01	4.20	4.31

Supplementary Table 5. SVP values of all reactant vibrational and translational modes with three transition states corresponding to O-H (TS1), C-H (TS2), and C-O (TS3) bond scissions.

Symmetry	Mode	Notes	SVP values		
			TS1	TS2	TS3
A'	OH stretching	ν_1	0.869	0.033	0.013
A'	CH_3 d-stretching	ν_2	0.003	0.178	0.023
A″	CH_3 d-stretching	ν_9	0.003	0.515	0.087
A'	CH_3 s-stretching	ν_3	0.005	0.508	0.104
A'	CH_3 d-deform	ν_4	0.062	0.511	0.317
A″	CH_3 d-deform	ν_{10}	0.007	0.232	0.018
A'	CH_3 s-deform	ν_5	0.013	0.105	0.126
A'	OH bend	ν_6	0.302	0.164	0.384
A″	CH_3 rock	ν_{11}	0.017	0.065	0.185
A'	CH_3 rock	ν_7	0.190	0.132	0.131
A'	CO stretching	ν_8	0.026	0.015	0.745
A″	torsion	ν_{12}	0.270	0.066	0.059
	translation x		0.000	0.103	0.024
	translation y		0.012	0.064	0.008
	translation z		0.121	0.100	0.242

Supplementary References

1. Kresse, G. & Furthmuller, J. Efficient iterative schemes for ab initio total-energy calculations using plane wave basis set. *Phys. Rev. B* **54**, 11169-11186 (1996).
2. Kresse, G. & Furthmuller, J. Efficiency of ab initio total energy calculations for metals and semiconductors using plane wave basis set. *Comp. Mater. Sci.* **6**, 15-50 (1996).
3. Perdew, J. P., Burke, K. & Ernzerhof, M. Generalized gradient approximation made simple. *Phys. Rev. Lett.* **77**, 3865-3868 (1996).
4. Jiří, K., David, R. B. & Angelos, M. Chemical accuracy for the van der Waals density functional. *J. Phys.: Condens. Matter* **22**, 022201 (2010).
5. Blochl, P. E. Project augmented-wave method. *Phys. Rev. B* **50**, 17953-17979 (1994).
6. Monkhorst, H. J. & Pack, J. D. Special points for Brillouin-zone integrations. *Phys. Rev. B* **13**, 5188-5192 (1976).
7. Lide, D. R. *CRC Handbook of Chemistry and Physics*, Internet Version 2005 edn. CRC Press (2005).
8. Henkelman, G., Uberuaga, B. P. & Jónsson, H. A climbing image nudged elastic band method for finding saddle points and minimum energy paths. *J. Chem. Phys.* **113**, 9901-9904 (2000).
9. Jiang, B. & Guo, H. Permutation invariant polynomial neural network approach to fitting potential energy surfaces. III. Molecule-surface interactions. *J. Chem. Phys.* **141**, 034109 (2014).
10. Jiang, B. & Guo, H. Six-dimensional quantum dynamics for dissociative chemisorption of H_2 and D_2 on Ag(111) on a permutation invariant potential energy surface. *Phys. Chem. Chem. Phys.* **16**, 24704-24715 (2014).
11. Jiang, B., Hu, X., Lin, S., Xie, D. & Guo, H. Six-dimensional quantum dynamics of dissociative chemisorption of H_2 on Co(0001) on an accurate global potential energy surface. *Phys. Chem. Chem. Phys.* **17**, 23346-23355 (2015).
12. Behler, J. Neural network potential-energy surfaces in chemistry: a tool for large-scale simulations. *Phys. Chem. Chem. Phys.* **13**, 17930-17955 (2011).
13. Raff, L. M., Komanduri, R., Hagan, M. & Bukkapatnam, S. T. S. *Neural Networks in Chemical Reaction Dynamics*. Oxford University Press (2012).
14. Jiang, B. & Guo, H. Permutation invariant polynomial neural network approach to fitting potential energy surfaces. *J. Chem. Phys.* **139**, 054112 (2013).
15. Li, J., Jiang, B. & Guo, H. Permutation invariant polynomial neural network approach to fitting potential energy surfaces. II. Four-atom systems. *J. Chem. Phys.* **139**, 204103 (2013).
16. Xie, Z. & Bowman, J. M. Permutationally invariant polynomial basis for molecular energy surface fitting via monomial symmetrization. *J. Chem. Theo. Comp.* **6**, 26–34 (2010).
17. Jiang, B., Yang, M., Xie, D. & Guo, H. Quantum dynamics of polyatomic dissociative chemisorption on transition metal surfaces: Mode specificity and bond selectivity. *Chem. Soc. Rev.* **45**, 3621-3640 (2016).

18. Zhang, Y.-l., Zhou, X.-y. & Jiang, B. Accelerating the Construction of Neural Network Potential Energy Surfaces: A Fast Hybrid Training Algorithm. *Chin. J. Chem. Phys.* **30**, 727-734 (2018).
19. Hu, X., Hase, W. L. & Pirraglia, T. Vectorization of the general Monte Carlo classical trajectory program VENUS. *J. Comp. Chem.* **12**, 1014-1024 (1991).
20. Jiang, B. & Guo, H. Relative efficacy of vibrational vs. translational excitation in promoting atom-diatom reactivity: Rigorous examination of Polanyi's rules and proposition of sudden vector projection (SVP) model. *J. Chem. Phys.* **138**, 234104 (2013).
21. Guo, H. & Jiang, B. The sudden vector projection model for reactivity: Mode specificity and bond selectivity made simple. *Acc. Chem. Res.* **47**, 3679-3685 (2014).
22. Polanyi, J. C. Some concepts in reaction dynamics. *Science* **236**, 680-690 (1987).
23. Jackson, B., Nattino, F. & Kroes, G.-J. Dissociative chemisorption of methane on metal surfaces: Tests of dynamical assumptions using quantum models and ab initio molecular dynamics. *J. Chem. Phys.* **141**, 054102 (2014).
